# Effect of Body Mass Index on the Disease Activity of Patients With Rheumatoid Arthritis in a Gender-Specific Manner and the Association of Respective Serum C-Reactive Protein Levels With the Body’s Inflammatory Status

**DOI:** 10.7759/cureus.9417

**Published:** 2020-07-27

**Authors:** Shumaila M Iqbal, Linda Burns, Joseph Grisanti

**Affiliations:** 1 Internal Medicine, University at Buffalo/Sisters of Charity Hospital, Buffalo, USA; 2 Rheumatology, Buffalo Rheumatology and Medicine, Buffalo, USA

**Keywords:** body mass index, rheumatoid arthritis, disease acitivity, c-reactive protein

## Abstract

Background

Current literature evaluating the effect of high body mass index (BMI) on the disease activity of patients with rheumatoid arthritis (RA) is mixed as some studies have shown a positive, linear relationship between BMI and disease activity while others have demonstrated an inverse correlation. Through this study, we have expanded the effect of BMI on disease activity in patients with RA. We have further expanded on whether BMI influences the disease activity depending on the gender being studied. Finally, we have studied whether there is a correlation between high BMI values and rising C-reactive protein (CRP) levels.

Methodology

This cross-sectional study was conducted at the Outpatient Clinical Department of Buffalo Rheumatology. The study was ethically approved by the Catholic Health Institutional Review Board. A total number of 451 patients’ clinical data was selected based on inclusion/exclusion criteria. The patients were divided into different BMI categories based on the guidelines of national obesity education initiative of the national heart, lung, and blood Institute. The following clinical parameters were studied: BMI, serum CRP level, and disease activity through routine assessment of patient index data questionnaire 3 (RAPID3). The minimum sample size (n = 358) was calculated via the world health organization sample size calculator. All data were entered and analyzed through Statistical Package for the Social Sciences (SPSS), version 16.0 (IBM Corp., Armonk, NY).

Results

Our study sample included 98 males and 353 females (22% and 78%, respectively). Collective data for both the genders showed significantly increased disease activity in RA patients with high BMI values (p = 0.04). When the data sets were categorized according to the two genders, it was noted that the aforementioned results remain significant for the females only (p = 0.02 for females and p = 0.57 for males). At all BMI values, mean RAPID3 scoring remained significantly higher for females as opposed to their male counterparts (p = 0.006). Mean serum CRP levels increased linearly with increasing BMI (p < 0.001); however, for the underweight patient population, mean CRP levels were the highest as compared to normal weight, overweight, moderately obese, and severely obese patients.

Conclusion

We conclude that the association between the BMI and the severity of disease remains elusive. High BMI values increase the risk of a pro-inflammatory state of the body due to higher serum CRP levels. Estimating the clinically significant benefit of this theory would require a large-scale clinical trial that would highlight the role of losing weight in improving the patients’ quality of life, pain control, and mortality.

## Introduction

The World Health Organization (WHO) defines obesity as excessive fat accumulation that poses a risk to physical health and body mass index (BMI) is a rough estimation of it [1]. Little is known regarding the effect of BMI on disease activity in rheumatoid arthritis (RA) patients. Some studies have shown high disease activity in overweight and obese patients with RA and women were found to be more prone to have increased disease activity levels due to high BMI in comparison to males [2,3]. Other studies have shown that high BMI in RA patients is protective against the amount of joint destruction, especially the small joints [4,5]

White adipose tissue is a source of a wide range of inflammatory proteins including tumor necrosis factor alpha, leptins, IL-1, IL-6, etc. Therefore, obesity creates a chronic inflammatory state in the body which in turn affects multiple organs systems including joints [6]. Plasma and synovial fluid leptin levels are observed to be elevated in patients with erosive and non-erosive RA and treatment with disease-modifying anti-rheumatic drugs (DMARDS) subsequently decreases serum leptin levels [7]. In some studies, though leptin levels were increased in obese patients, the correlation with the disease activity was not observed [8,9]. Adiposity is also found to be independently associated with C-reactive protein (CRP) levels in women with RA. The increased concentration of CRP provides a good estimation of RA disease activity when it is used as a surrogate for systemic inflammation [10].

Through this cross-sectional study, we have expanded upon the relationship between BMI and disease activity in RA. We have further expanded on whether BMI influences the disease activity depending on the gender being studied. Finally, we have studied whether there is a correlation between high BMI values and rising CRP levels.

## Materials and methods

Ethical review and study setting

The retrospective study was approved ethically by the institutional review board of Catholic Health System and was conducted in the outpatient, clinic-based Department of Buffalo Rheumatology and Medicine.

Sample technique and duration of study

The sampling technique used was convenient sampling. The duration of the study was six months. It was conducted between November 2018 till June 2019.

Sample size

The sample size was calculated via WHO sample size calculator with the following parameters: confidence level: 95%, margin of error/confidence interval (CI): 5%, and population: 5161 [2]. The minimum sample size calculated was 358.

Inclusion/exclusion Criteria

Male and female RA patients 18 years or older who have attended the outpatient clinic were selected as study subjects. The study subjects should have RA diagnosed according to the 2010 American College of Rheumatology/European League against rheumatism RA classification criteria.

Studied clinical parameters

Disease Activity

The patient’s disease activity was evaluated through self-reported routine assessment of patient index data 3 (RAPID3) questionnaire which is an index found within a multi-dimensional health assessment questionnaire (MDHAQ) for routine clinical care. It is a simple, reliable, and valid tool to assess disease activity [11]. It is composed of three self-reported scores for physical functionality, pain, and the patient’s global estimate of his or her health; each section is scored from 0-10, for a total of 0-30 points [12]. These points are then entered as the patients’ cumulative score for RAPID3. The grading of disease activity on the basis of cumulative score is: near remission (score: 1-3), low severity (score: 4-6), moderate severity (score: 7-12), and high severity (score 13-30).

Body Mass Index

The BMI value was evaluated by the formula: the patient’s weight (in kilograms) divided by the square of the height (in meters). BMI values were categorized according to the widely used guideline of national obesity education initiative of the national heart, lung, and blood Institute as: underweight (<18.5 kg/m2), normal (18.5-24.9 kg/m2), overweight (25-29.9 kg/m2), moderately obese (30-34.9 kg/m2), severely obese (35-39.9 kg/m2) and very severely obese (>40kg/m2) [13]

C-Reactive Protein

Serum CRP levels were determined through laboratory serum blood testing.

Gender

Determination of gender was made through the patient’s electronic medical record (EMR).

Sample collection

A total number of 2000 patients with RA being treated at Buffalo Rheumatology were initially enrolled. Out of those patients, only 451 patients’ clinical data were selected for the research purpose based on inclusion/exclusion criteria and the availability of complete clinical data from their subsequent clinic visits which included BMI, gender, serum CRP levels, and scoring of the RAPID3 questionnaire. Clinical data for the aforementioned variables were achieved by reviewing the patients’ chart through electronic medical record. The clinical data were recorded and analyzed through Statistical Package for the Social Sciences (SPSS), version 16.0 (IBM Corp., Armonk, NY). Through mean values, Pearson’s chi-square probability testing, independent sample t-test, and pairwise comparison, the association of the patients’ BMI with disease activity along with the possible association with the patients’ CRP levels was evaluated in males and females.

## Results

Our study included 353 females and 98 males (78% and 22%), respectively. The baseline data of the recruited patients are shown in Table 1.

**Table 1:** Baseline data for recruited patients. Through independent sample t-test, p values for the mean within a group were calculated. BMI = body mass index; RAPID3 = routine assessment of patient index data 3; CRP = C-reactive protein; p value = Pearson’s chi-square probability.

Characteristics	Males (n = 98) (mean + SD)	Females (n = 353) (mean + SD)	P values
Age	63.4 + 13.1	61.3 + 14.4	0.192
BMI	30.7 + 7.8	30.9 + 9.5	0.869
RAPID3 Score	8.3 + 6.6	10.5 + 7.3	0.006
CRP	3.2 + 5.5	4.1 + 7.5	0.291

Comparison of mean routine assessment of patient index data 3 scores among different categories of BMI

**Figure 1:**
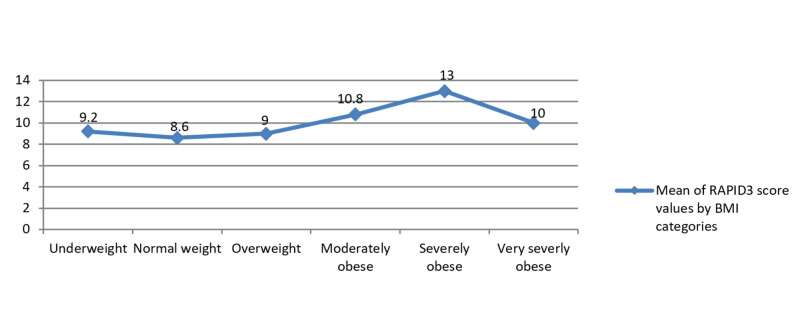
Plots showing mean RAPID3 score values with BMI categories mentioned on x-axis. BMI = body mass index; RAPID3 = routine assessment of patient index data 3.

Patient’s BMI was found to be significantly associated with disease activity level (pearson’s chi-square probability testing: p = 0.04). The mean score for the RAPID3 questionnaire in different BMI categories increased with increasing BMI (Figure 1).

However, in patients with very severe obesity, the mean scores for disease activity were low as compared to ones with severe obesity. Additionally, the patients in the underweight category demonstrated a higher mean for RAPID scores as compared to those in the normal and overweight category. A pairwise comparison using the turkey adjustment showed that mean RAPID scores for obese patients were significantly different from normal-weight patients (4.3 times, p = 0.006, 95% CI: -7.83, -0.83) and overweight patients (3.9 times, p = 0.011, 95% CI: -7.29, -0.583).

Gender-based differences in disease activity

**Figure 2:**
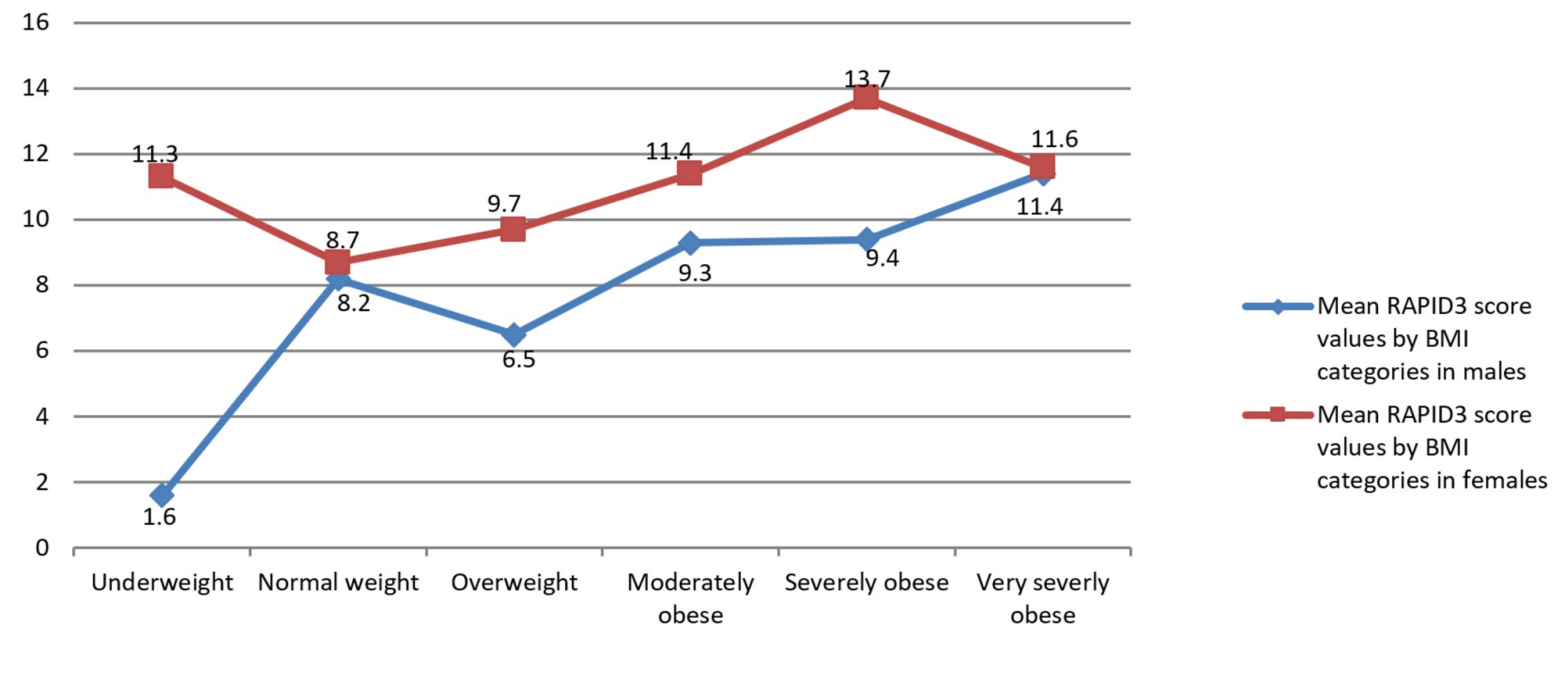
Plots are mean values for RAPID3 scores with BMI mentioned on x-axis. BMI = body mass index; RAPID3 = routine assessment of patient index data 3.

When evaluated in individual genders, only the female gender was found to have disease activity significantly associated with increasing BMI (Pearson’s chi-square probability test: p = 0.02). The results remained non-significant for the male gender (Pearson’s chi-square probability test: p = 0.573). Mean values for RAPID3 scores in individual gender have been mentioned in Figure 2 by BMI categories.

For each corresponding BMI category, the mean RAPID3 score values were statistically higher for females as compared to males (independent samples t-test: p = 0.006, 95% CI -3.8, -0.65) (Figure 2). The exception to this was a group of male and female with very severe obesity (BMI>40kg/m2) where both genders share more or less the same mean for RAPID3 scores (figure 2).

Mean scores for individual components of RAPID3

**Figure 3:**
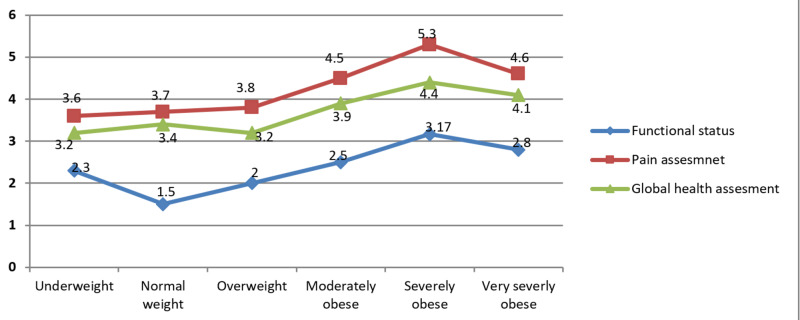
Plots are mean scores for individual component of RAPID3 with BMI categories mentioned on the x-axis. BMI = body mass index; RAPID3 = routine assessment of patient index data 3.

For all three components of RAPID3, mean scores increased linearly with an increase in BMI. However a decline in mean scores was observed for very severely obese patients when compared to the mean scores for severely obese patients (Figure 3).

Pain was graded highest as compared to the other two components of RAPID3 by patients in all BMI categories followed by global health assessment and functional status. A pairwise comparison using the turkey adjustment showed significantly higher pain grading in severely obese patients in comparison to normal weight (1.6 times, p = 0.24, 95% CI: -3.08, -0.13) and overweight patients (1.5 times, p = 0.29, 95% CI: -2.91, -0.09). By the same comparison using the turkey adjustment, functional status was significantly better in normal weight patients as compared to moderately obese (0.96 times, p = 0.22, 95% CI: -1.84, -0.08) and severely obese patients (1.6 times, p = 0.00, 95% CI: -3.08, -0.13).

Serum CRP and BMI

**Figure 4:**
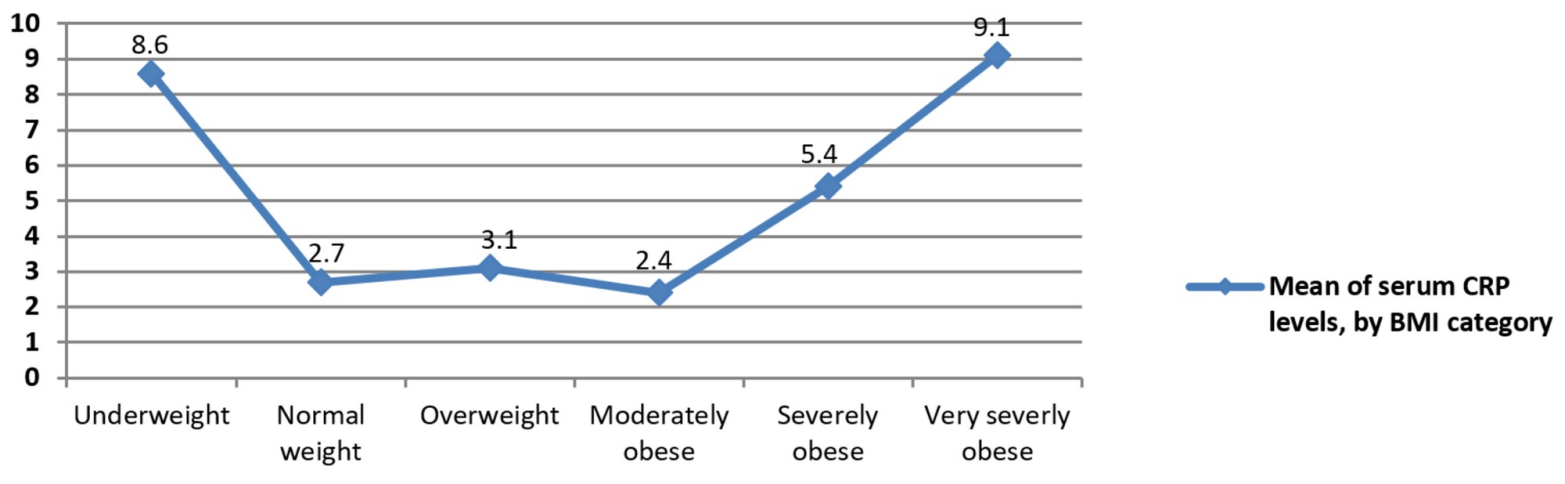
Plots are mean serum CRP values with BMI categories mentioned on the x-axis BMI = body mass index; CRP = C-reactive protein.

As per the data analysis, the highest mean values for CRP were witnessed at the two extremes of the BMI categories, the underweight and very severely obese patients (8.6 + 15.9 SD and 9.1 + 10.1) respectively (Figure 4).

Overall a significant association was found between the BMI and CRP values (Pearson’s chi-square testing: p < 0.001). Pairwise comparison using the turkey adjustment demonstrated that very severely obese patients have mean CRP levels significantly higher than normal weight (6.38 times, p < 0.001, 95% CI: 0.03, 9.73), overweight (5.94 times, p =<0.001, 95% CI: 2.73, 9.41), and moderately obese patients (6.71 times, p < 0.001, 95% CI: 3.31, 10.1), respectively.

Serum CRP and disease activity

**Figure 5:**
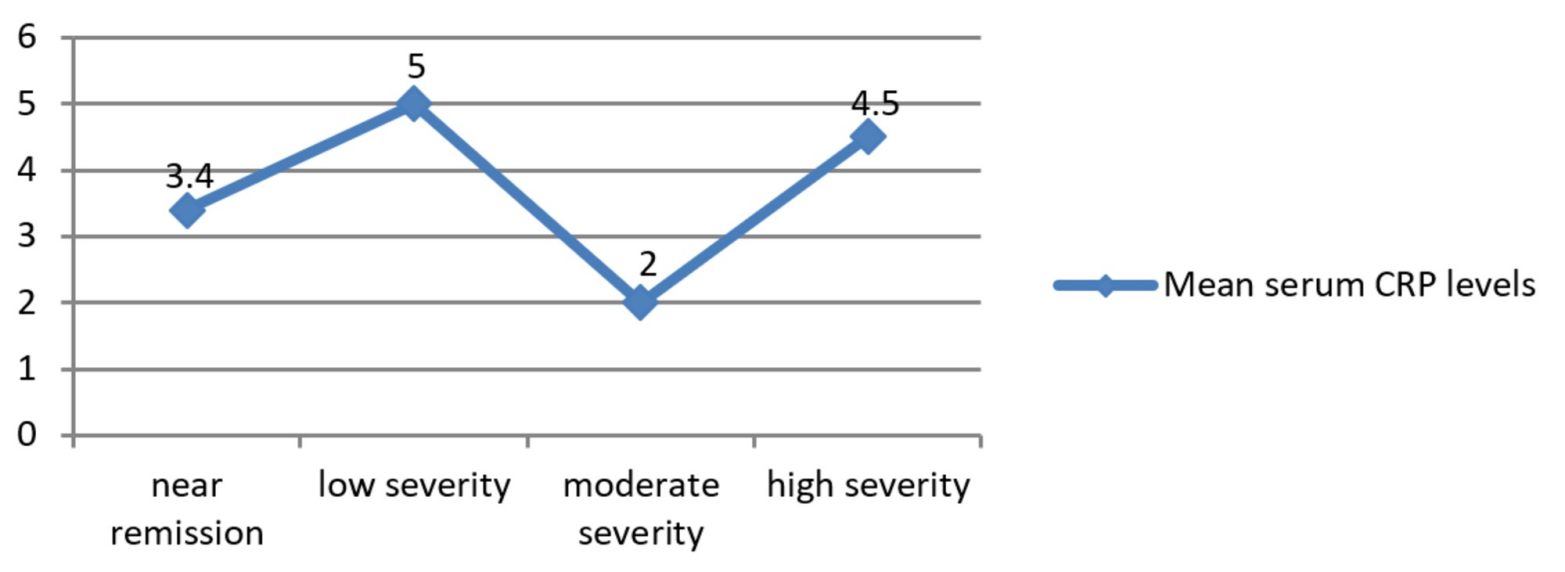
Plots are mean serum CRP levels with severity of disease activity level on x axis. CRP = C-reactive protein.

The association of serum CRP level and disease activity as per Pearson’s chi-square probability was non-significant (p = 0.222). The highest serum CRP levels were observed in patients having low disease severity and then subsequent higher serum CRP levels in patients with high disease activity, near remission, and moderate disease severity, respectively (Figure 5).

## Discussion

The medical literature has shown that there is a higher prevalence and incidence of RA in obese and overweight population [14]. However, there might be differences in this association based on confounding factors like the patient population, the duration of disease, and the type of treatment offered at the time of evaluation. A meta-analysis by Zhou and Sun on 353,948 patients showed that there was a higher incidence of RA in overweight and obese populations with an odds ratio of 1.08 (95% CI: 1.00, 1.15) and 1.32 (95% CI: 1.11, 1.54), respectively [15]. Supporting this, another cohort study performed on 813 Minnesotans also demonstrated that the increasing prevalence of obesity is associated with a modest increase in the incidence of RA [16]. Baseline clinical data for our study also shows that 78.6% of our recruited patient population falls in the range of having a BMI of >25kg/m2 (37.5% overweight and 41.1% obese patient population).

Our retrospective study was successful in demonstrating that there was an increase in disease activity in RA patients with increasing BMI; moreover, the RAPID3 scores were recorded to be the highest in severely and very severely obese patients. These results were comparable to the large QUEST-RA study involving 1561 patients with RA [2]. The exact mechanism for obesity-inducing the pro-inflammatory state and oxidative stress to one’s body is unknown. However, it is well established that macronutrients in adipose tissue increase the release of pro-inflammatory mediators like TNF-alpha and IL-6 (TNF-alpha and IL-6 ) and decrease adiponectin production [17,18]. IL-6 in turn increases CRP production in the liver which binds to phosphocholine in the presence of ionized calcium. This ligand-bound CRP, in turn, activates complement and enables its binding to Fc receptors of the antibodies. It thus potentiates antibody-mediated clearance of pathogenic antigens. Less well understood is the CRP’s ability to bind autoantigens and its presumed capacity to clear apoptotic cells [19]. If this theory is considered, then it is plausible to imagine that the greater the amount of adipose tissue in the body, the greater the CRP mediated disease activity in RA patients. Our study results support the aforementioned idea and also demonstrates a linear progression in the mean CRP values and increasing BMI (18.5 kg/m2 to >40 kg/m2).

A striking finding was also observed in the underweight patient population that is, the mean CRP levels were higher in underweight patients as compared to normal weight, overweight, moderately and severely obese patient population. Interestingly, despite having higher mean serum CRP values, the reported disease activity by underweight patients doesn’t differ significantly from disease activity in normal weight, overweight, moderately, and severely obese patients (as per pairwise comparison using the turkey adjustment). One possible explanation for this discrepancy could be the subjective assessment of the disease status by the underweight patient population while filling out the RAPID3 questionnaire. The second possible explanation could be related to the results of the study performed in murine models. It demonstrated that the increased CRP is protective against autoimmune phenomenon [19]. The conjecture that the CRP levels pose protective effects in halting the autoimmune disease process needs to be further studied to find out about their significance in the obese and overweight population.

Our study also showed gender-specific differences in disease activity of RA patients. The association of the worsening of disease with an increase in BMI was found to be significant in the female study population. This might be due to the fact that the study sample had more female candidates; the ratio of male to female was close to 1:3. We inferred that if we had more male candidates in our study group, we would have had the power to find a more clinically significant association of disease activity and increasing BMI. The other highlighted finding from our study was, at any BMI, the reported disease activity was higher in females as compared to males. The reason could be the difference in the amount of fat a male and female can have with exact similar BMI. Males have more muscle mass contributing towards BMI as compared to females who have more fat and less muscle mass. This could explain an increase in the pro-inflammatory state responsible for higher disease activity in females as compared to males at any BMI. We also assume that in very severe obese male and female (BMI >40kg/m2), there might be nullification of this phenomenon when the male patients start to accumulate more cutaneous fat rather than visceral as noticed in our study on the equalization of disease activity for very severely obese male and female [2].

The study has many limitations which the authors would like to address here. We believe that, if we were able to increase the number of recruited patients for the final data analysis, we might have been able to see if the association of disease activity in RA with increased BMI has more significance than our current results show. Secondly, as we have already addressed, increasing the number of male patients in our study group might result in the significant influence of increasing BMI on disease activity in male patients with RA. We would also like to address, the utilization of patients’ reported estimates of their disease activity through RAPID3 questionnaire is subjective and thus rely on the patients’ own judgment to their disease would be influential to the study results. Another limitation is the utilization of BMI to determine the approximation of total body fat in patients with RA due to unavailability of investigative tools required for accurate measurement of total body fat in our clinic setting. Moreover, we were not certain as to how much percentage of fat is contributing to the total BMI in males and females, respectively. The final limitation was that most of the patients selected for the study were under the treatment of DMARDS during the study period. Therefore, the medications could have exerted an effect on the patients’ BMI [20-23].

Authors would also like to disclose here that the abstract of this article has been presented at 2019 American College of Rheumatology/The Association of Rheumatology Professionals Annual Meeting [24].

## Conclusions

We conclude that the association between the BMI and the severity of disease remains elusive. Estimating the clinically significant benefit of these theories would require a large-scale clinical trial that would highlight the role of losing weight in improving the patients’ quality of life, pain control, and mortality. Our findings are crucial because obesity doesn’t only play a role in increasing the pro-inflammatory cytokines. It also increases the cardiovascular events in the patients. Moreover, the life span of the RA patients is less than that of the general population. Therefore, more studies would provide life-long benefits to these patients. At this point in time, we would like to recommend educating patients on the importance of weight loss to better their quality of life. This strategy might prove to lessen the disease activity of RA as well as providing the protection against cardiovascular outcomes.
